# Physical activity modulates inhibitory control: EEG insights from P300 and theta oscillations

**DOI:** 10.3389/fnhum.2025.1591458

**Published:** 2025-06-06

**Authors:** Liqing Liu, Yan Hao, Zhihui Zhao, Qing Liu

**Affiliations:** ^1^Faculty of Psychology, Tianjin Normal University, Tianjin, China; ^2^Key Research Base of Humanities and Social Sciences of the Ministry of Education, Academy of Psychology and Behavior, Tianjin Normal University, Tianjin, China; ^3^Tianjin Key Laboratory of Student Mental Health and Intelligence Assessment, Tianjin, China

**Keywords:** physical activity, inhibitory control, EEG, amplitude, oscillations

## Abstract

**Introduction:**

Previous studies have mainly focused on the effects of exercise on inhibitory control, but the impact of daily physical activity has not been thoroughly examined. This study aims to investigate how physical activity affects inhibitory control functions and the underlying neural mechanisms involved.

**Methods:**

We examined 36 adults, divided into two groups based on their daily physical activity levels: a high physical activity level (HPAL) group consisting of 18 participants and a low physical activity level (LPAL) group, also with 18 participants. Physical activity levels were measured using the International Physical Activity Scale. Neural dynamics were recorded through electroencephalography (EEG) as participants performed a modified Flanker task to assess their inhibitory control abilities.

**Results:**

Our results indicate that physical activity has a significant impact on inhibitory control. Individuals with high levels of physical activity showed significantly larger P300 amplitudes compared to those in the low physical activity group. Additionally, we observed enhanced theta and alpha oscillatory power in the HPAL group when compared to the LPAL group. Furthermore, we found a positive association between higher levels of physical activity among participants and increased amplitudes of P300 as well as theta oscillations.

**Discussion:**

Our findings provide electrophysiological evidence that physical activity strengthens inhibitory control through the enhancement of conflict-related P300 responses and synchronization of prefrontal theta activity, suggesting improved neural efficiency in managing cognitive interference.

## 1 Introduction

Inhibitory control is a crucial component of executive function and has been a primary research focus in cognitive neuroscience. It is the ability to suppress habitual or dominant responses, manage attentional resources, and prevent interference from irrelevant information (Miyake et al., 2000). Inhibitory control can enhance cognitive processing efficiency by improving concentration and reducing susceptibility to external distractions. Improvements in inhibitory control can positively impact decision-making and behavioral regulation in daily life and the workplace. Previous studies have explored various intervention methods to enhance inhibitory control, such as computerized tasks, video game training, and physical exercise interventions, each showing varying degrees of effectiveness (Bai and Zhou, 2022). Exercise interventions have emerged as the most widely applicable approach among these methods.

Physical activity (PA) was initially defined by Caspersen et al. (1985) as “any bodily movement produced by skeletal muscles that results in energy expenditure,” which encompasses various forms of movement, including exercise training, leisure and entertainment activities, recreational activities, occupational tasks, and household chores. Previous studies have identified a significant positive relationship between PA and executive function. Research has shown that physical activity benefits cognitive abilities in young people, especially in terms of executive inhibitory control (Salas-Gomez et al., 2020). Although there is limited research specifically focusing on the effects of physical activity on inhibitory control, numerous studies have examined the impact of exercise on this function from various angles. For instance, Wang et al. (2013) examined the effects of exercise intensity by randomly assigning 80 participants into four groups: control group, low-intensity group, moderate-intensity group, and high-intensity group. They found participants in the high-intensity group exhibited impaired performance in inhibitory control. In contrast, Tian et al. (2021) reported that high-intensity interval training (HIIT) could improve the inhibitory control function that lasted for at least 90 min. Moreover, a recent meta-analysis by Wu et al. (2023) suggested that both HIIT and moderate exercise can lead to similar improvements in inhibitory control. Beyond the intensity of exercise, researchers have also investigated the effects of duration of physical activity. Studies have shown that both acute exercise (Davranche et al., 2009; Hatch et al., 2021; Won et al., 2019; Yu et al., 2020) and long-term training (Tempest et al., 2017; Wu et al., 2024) could affect inhibitory control function. Behavioral effects are generally observed as changed accuracy (ACC) and reaction times (RT), while the neurophysiological effects include changes in event-related potential (ERP) components such as P300 (Hillman et al., 2004; Scharinger et al., 2017) and N200 (Groom and Cragg, 2015; Huster et al., 2013).

Previous studies have investigated the neural mechanisms underlying the effects of exercise on inhibitory control functions. For example, using a go/no-go paradigm, Zheng (2022) demonstrated that 6 months of brisk walking resulted in an increased P3 amplitude and a decreased latency in participants who exercised compared to the control group. This finding is consistent with earlier studies (Kao et al., 2017; Olson et al., 2016; Xie et al., 2024). Additionally, prior research has indicated that the N200 component is linked to inhibitory function, showing decreased amplitude and latency following exercise (Chueh et al., 2023; Drollette et al., 2014; Wen et al., 2022). Emerging evidence also highlights the role of oscillatory dynamics in inhibitory control, particularly the theta (Cavanagh and Frank, 2014; Chmielewski et al., 2016; Nigbur et al., 2011) and alpha oscillations (Duprez et al., 2020). Griggs et al. (2023) found enhanced theta power during flanker tasks following acute exercise, supporting earlier findings (Hong et al., 2018; Wang et al., 2020). Similarly, increases in alpha power resulting from exercise have also been documented (Chaire et al., 2020; Ciria et al., 2018).

Alongside exercise training, physical activity includes various types of movement, such as leisure and entertainment activities, recreational tasks, occupational duties, and household chores. Previous studies primarily examined the effects of exercise on inhibitory control, while the impacts of daily physical activity have not been sufficiently explored. For instance, behavioral experiments conducted by Salas-Gomez et al. (2020) demonstrated the positive effects of PA on executive function. Similarly, Zhao et al. (2024) found significant correlations between PA levels and the executive control function of college students. However, conflicting findings exist in the literature (Ho et al., 2018; Stenling et al., 2021), and current evidence mainly relies on psychometric assessments rather than neurophysiological measures. This study aims to bridge these gaps by utilizing EEG technology to investigate the effects of PA on inhibitory control through behavioral analysis, event-related potentials (ERPs), and neural oscillation measurements. Our approach seeks to clarify the neurocognitive mechanisms involved in the effects of PA, providing novel insights into the relationship between daily activity and brain function.

## 2 Methods and materials

### 2.1 Participants

This study utilized G*power 3.1 software (Faul et al., 2009) to estimate the required experimental sample size in a mixed experiment design, setting the significance level (*α*) at 0.05 and the effect size at 0.25 while using the default value for the correlation between repeated measures and non-sphericity correction. It was anticipated that a total sample of at least 24 participants would be necessary to achieve an 80% statistical power level. However, to account for uncertainties with the statistical power, the final sample size was adjusted to 40. The inclusion criteria for participants were as follows: participants had to be aged 18–35 years, all were right-handed, none had diseases related to movement or cognition, all had normal or corrected-to-normal vision, and their Beck Depression Inventory scores were 13 points or lower. All participants volunteered for the study and signed an informed consent form. The study was approved by the Institutional Review Board of Tianjin Normal University, China. The participants were divided into two groups based on their physical activity levels: a high physical activity level (HPAL) group and a low physical activity level (LPAL) group, according to the scoring criteria of the long version of the International Physical Activity Scale (IPAQ) (Craig et al., 2003). Some participants’ data were excluded due to quality issues, resulting in 18 valid participants in each group: 9 males in the HPAL group and 7 males in the LPAL group. There were no significant differences in gender (*p* = 0.735, Cramer’s V = 0.056) between the two groups. The BMI values were comparable between the HPAL group (21.6 ± 3.7) and LPAL group (21 ± 2.2) (*p* = 0.057, Cohen’s d = 0.0148).

### 2.2 Experimental procedure

**Figure 1:**
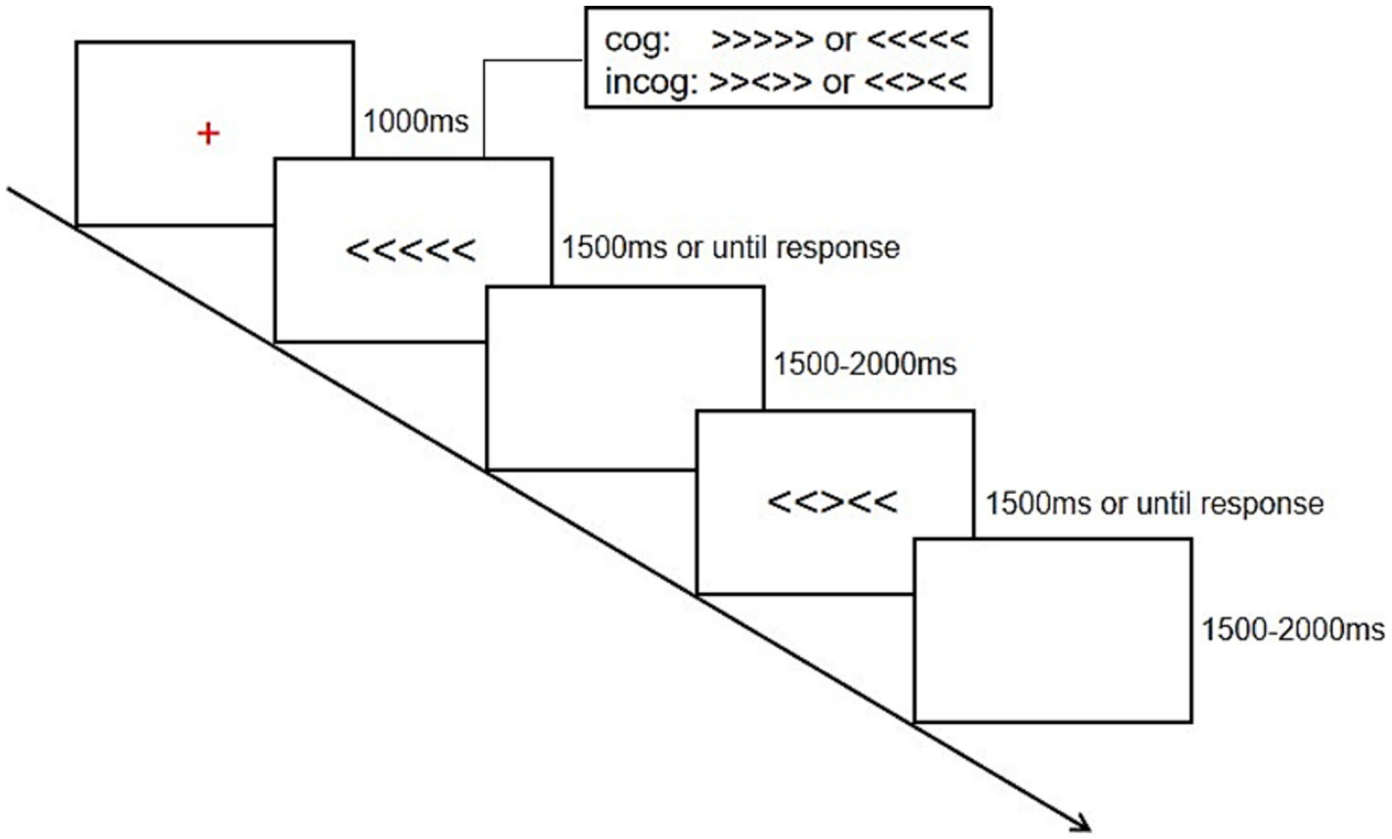
The flowchart of the experimental paradigm. cog indicates the consistent condition; incog indicates the inconsistent condition.

This experiment employs a mixed experimental design. It consists of two groups (HPAL and LPAL groups) and two conditions (consistent and inconsistent conditions). The experimental task is based on a Flanker task, which comprises two types of conditions: consistent and inconsistent. The flowchart of the experiment is illustrated in Figure 1. At the beginning of the experiment, a fixation point is displayed for 1,000 milliseconds, followed by the target stimulus presented for 1,500 milliseconds. The target stimuli differ in consistent and inconsistent conditions. In the consistent condition, the target stimuli are either <<<<< or >>>>>, while in the inconsistent condition, the target stimuli are <<> < < or > > <>>, where the direction of the middle arrow contrasts with others. Participants are instructed to quickly press the “F” button if the middle arrow points left and the “J” button if it points right, responding as rapidly as possible. The target stimuli in both conditions are presented in a random order. The target stimuli will disappear once the participants respond. After each response, a blank screen appears for 1,500 to 2,000 milliseconds. The entire task consists of 2 blocks, each containing 80 trials—40 in the consistent condition and 40 in the inconsistent condition.

### 2.3 Data collection and processing

E-Prime 3.0 software was utilized to acquire reaction times and accuracy during the task, while SPSS 26.0 was employed for the statistical analysis of the data. EEG data were collected using the Neuroscan Curry 8 system and a 64-electrode EEG cap. During data collection, a default reference electrode was used. Two horizontal electrooculogram electrodes (HEOG) were positioned 1 centimeter from the left and right sides of the participant’s eyes, and two vertical electrooculogram electrodes (VEOG) were placed 1 centimeter above and below the left eye, oriented perpendicularly to the pupil. The impedance for all electrodes was maintained below 10 kΩ, and the data sampling rate was set to 500 Hz.

The preprocessing steps are as follows: remove electrodes (CB1, CB2, VEO, HEO); apply a bandpass filter with a frequency range of 0.1 to 40 Hz; downsample the data to 250 Hz; epoch the data by using the target stimulus as the zero point, selecting a time window from 500 ms before to 2000 ms after the target stimulus; identify bad electrodes and interpolate their values; re-reference the data using the bilateral mastoids (M1 and M2) as reference electrodes; conduct independent component analysis (ICA) to remove eye movement artifacts using the ADJUST1.1.1 plug-in; eliminate extreme values that are greater than ±100 μV; perform baseline correction using the 500 ms preceding the target stimulus as the baseline; average the trials across all conditions for each participant.

For the ERP analyses, the primary focus was on the components from N200 and P300, based on previous research. The N200 component utilized the Fz and Cz electrodes, while the P300 component involved the Fz, Cz, and Pz electrodes as regions of interest (Van’t Ent, 2002). The amplitudes of N200 and P300 were calculated by averaging the 50-ms time window around the peak of each component for each group. For the time-frequency analysis, a self-written MATLAB script was utilized to average the frequencies of the theta and alpha oscillations, with the stimulus presentation marked as the zero point. The time range of 0–1 s was selected to observe the spectral energy across each frequency band. To process the data from all participants, trials, conditions, electrodes, and across a frequency range of 1–40 Hz, a 300 ms time window with a 1 Hz step was applied. This data was then transformed into the time-frequency domain using the short-time Fourier transform method. For the statistical analysis, as the data fail to meet the normality assumptions based on the results of Kolmogorov–Smirnov (K-S) test (*p* < 0.01), we employed permutation test for two-way ANOVA (10,000 randomizations) for data analysis.

## 3 Results

### 3.1 Behavioral results

Results of statistical analyses on behavioral measures were shown in Table 1. The results of RT showed that the main effect of the group was not significant [*F* (1, 34) = 2.82, *p* = 0.0964]. However, the main effect of the condition was significant [*F* (1, 34) = 25.44, *p* < 0.001, *η* ^2^ *p* = 0.43]. Participants had significantly faster reaction times in the consistent condition (421.432 ± 10.106 ms) compared to the inconsistent condition (496.374 ± 10.893 ms). Additionally, the interaction between group and condition was not significant [*F* (1, 34) = 0.01, *p* = 0.9223].

**Table 1:** Results of statistical analyses on behavioral measures.

Variable	Variance		$\bar{x}$ ±s	*F*	*p*
RT	Group	HPAL	446.420 ± 14.565	1.469	0.234
		LPAL	471.386 ± 14.565		
	Condition	cog	421.432 ± 10.106	325.286	***
		incog	496.374 ± 10.893		
	Group * condition			0.132	0.719
ACC	Group	HPAL	0.974 ± 0.006	0.009	0.927
		LPAL	0.975 ± 0.006		
	Condition	cog	0.995 ± 0.002	30.898	***
		incog	0.953 ± 0.008		
	Group * condition			0.034	0.855

* indicates *p* < 0.05; ** indicates *p* < 0.01; *** indicates *p* < 0.001; RT, reaction times; ACC, accuracy; HPAL, high physical activity level; LPAL, low physical activity level; cog: consistent condition; incog, inconsistent condition.

In the ACC analysis, the main effect of the group was not significant [*F* (1, 34) = 0.01, *p* = 0.9185]. However, the main effect of the condition was significant [*F* (1, 34) = 25.56, *p* < 0.001, *η* ^2^ *p* = 0.43]. The accuracy rate for the consistent condition (0.995 ± 0.002) was significantly higher than that for the inconsistent condition (0.953 ± 0.008). Additionally, the interaction between group and condition was not significant [*F* (1, 34) = 0.03, *p* = 0.8728].

### 3.2 P300/N200 components

**Figure 2:**
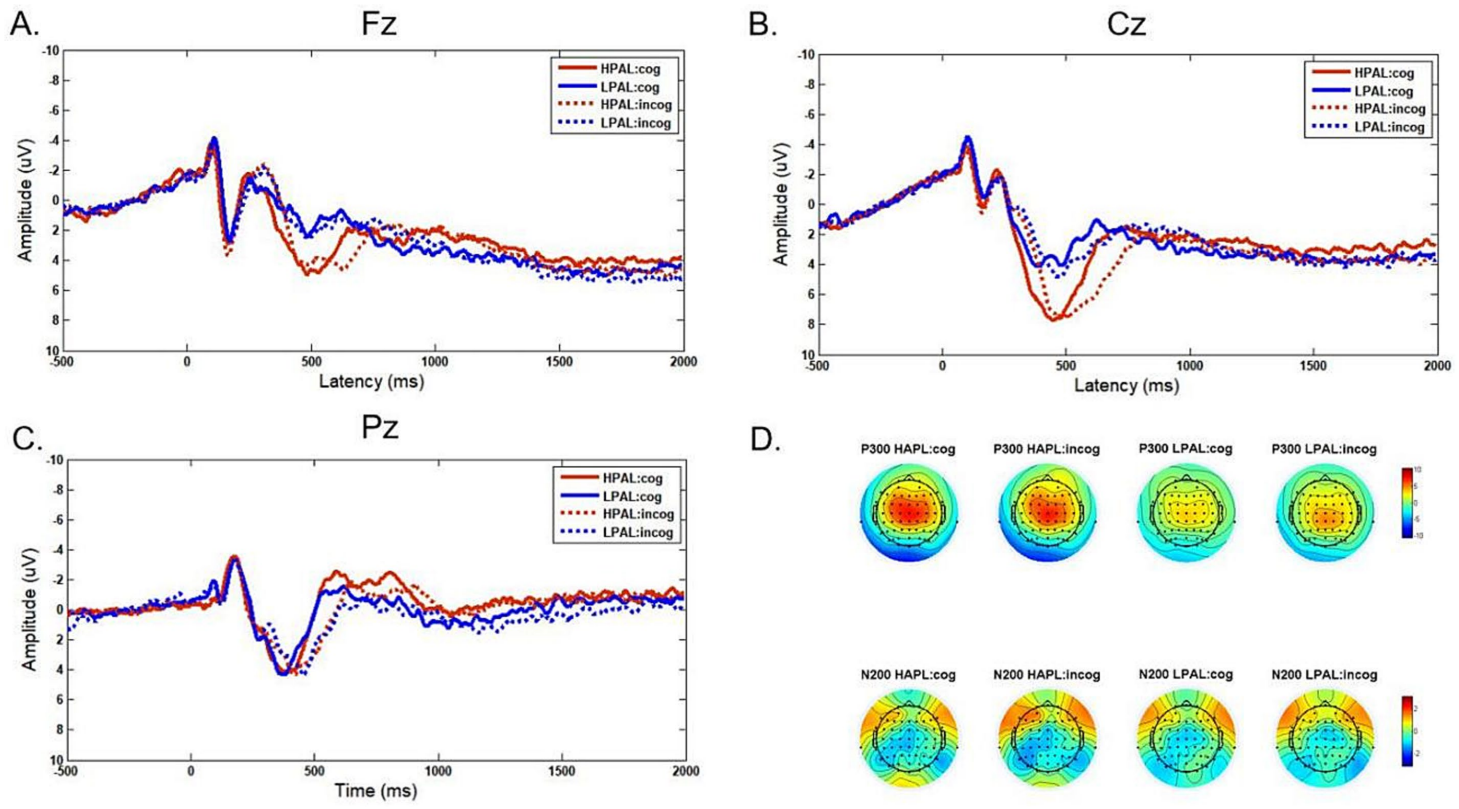
P300 and N200 components in consistent and inconsistent conditions in both HPAL and LPAL groups at the Fz **(A)**, Cz **(B)** and Pz **(C)** electrodes, alongside with the topographical maps **(D)**. HPAL, cog indicates consistent condition of high physical level group; LPAL, cog indicates consistent condition of low physical level group; HPAL, incog indicates inconsistent condition of high physical level group; LPAL: incog indicates inconsistent condition of low physical level group.

The results for the Fz electrode indicated a significant main effect of the group on the average amplitude of P300 [*F* (1, 34) = 8.15, *p* < 0.001, *η* ^2^ *p* = 0.19]. However, the main effect of the condition was not significant [*F* (1, 34) = 0.001, *p* = 0.9706]. Additionally, the interaction between group and condition was not significant [*F* (1, 34) = 0.27, *p* = 0.6119]. As it was shown in Figure 2A, the HPAL group exhibited a larger amplitude than the LPAL group in both consistent and inconsistent conditions. The topographical maps from 430 ms to 530 ms are presented in the upper panel of Figure 2D. The power in the HPAL group was significantly higher in the frontal and central regions compared to the LPAL group.

On the Cz electrode, the main effect of the group was significant [*F* (1, 34) = 17.44, *p* < 0.001, *η^2^p* = 0.34]. Compared to participants with a high level of physical activity, participants with low levels of activity had smaller P300 amplitudes. However, the main effect of the condition [*F* (1, 34) = 0.25, *p* = 0.6230] and the interaction between the group and condition are not significant [*F* (1, 34) = 0.43, *p* = 0.5090]. The detailed results were shown in Figure 2B. No significant results were found on P300 amplitude on the Pz electrode.

The amplitude of the N200 component on the Fz and Cz electrodes was examined in the study. The main effects of the group and the condition, as well as their interaction, did not reach statistically significant levels. The N200 component on the Fz and Cz electrodes for both consistent and inconsistent conditions in the two groups is illustrated in Figures 2A,B, respectively. Additionally, the topographical maps of the N200 component from 200 ms to 360 ms are shown in the lower panel of Figure 2D.

### 3.3 Theta oscillations

**Figure 3:**
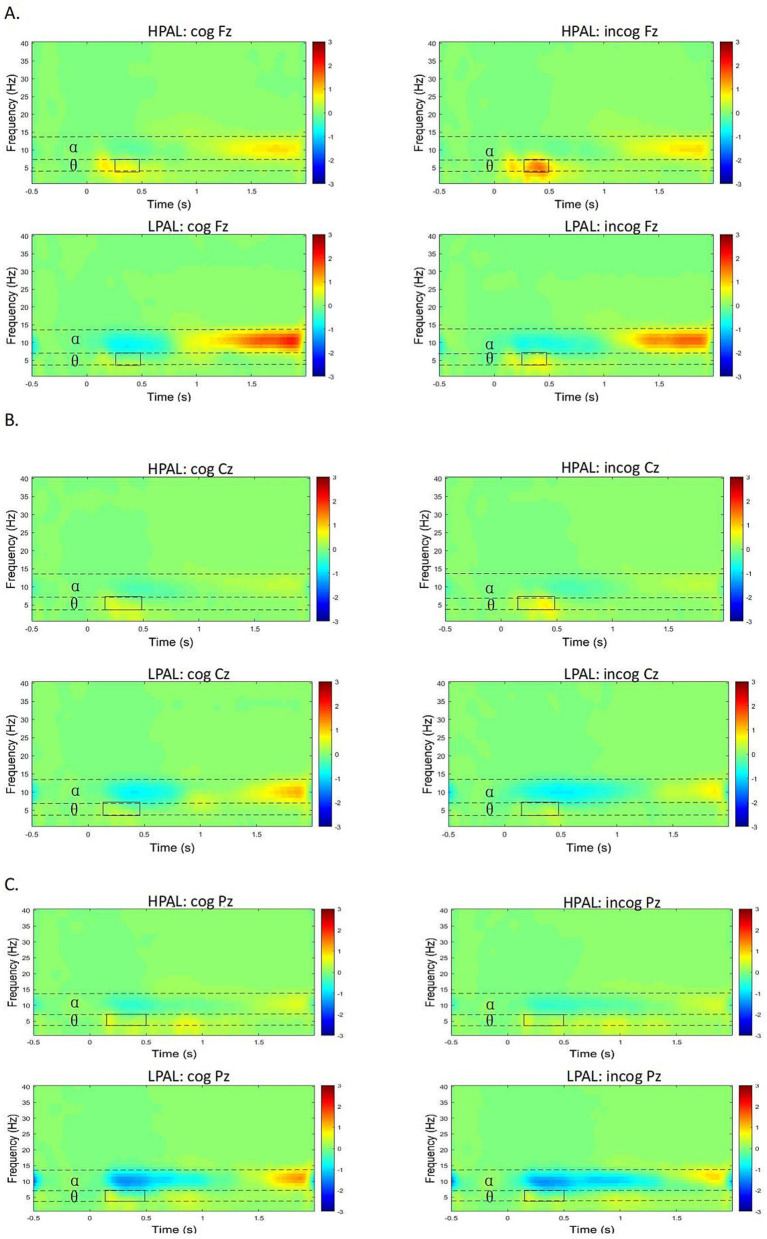
Neural oscillations across various frequency bands under different conditions and among different groups at the Fz **(A)**, Cz **(B)** and Pz **(C)** electrodes. HPAL, cog indicates consistent condition of high physical level group; LPAL, cog indicates consistent condition of low physical level group; HPAL, incog indicates inconsistent condition of high physical level group; LPAL: incog indicates inconsistent condition of low physical level group.

Time-frequency plots for the Fz, Cz, and Pz electrodes are presented in Figures 3A–C, respectively. Overall, the task performance resulted in increased theta and decreased alpha oscillatory activity. For the statistical analysis, a permutation test for a two-way ANOVA was conducted. For the Fz electrode, the analysis of theta oscillations revealed a significant main effect of group during the following time ranges: 0–200 ms [*F* (1, 34) = 9.63, *p* < 0.001, *η^2^p* = 0.22], 200–500 ms [*F* (1,34) = 14.65, *p* < 0.001, *η^2^p* = 0.30], and 500–1,000 ms [*F* (1, 34) = 5.86, *p* = 0.0133, *η^2^p* = 0.15]. As illustrated in Figure 3A, theta oscillations were significantly greater in the HPAL group than in the LPAL group. Additionally, a significant main effect of condition was found in the 200–500 ms time window [*F* (1, 34) = 6.79, *p* = 0.0102, *η^2^p* = 0.17], indicating that theta oscillation activity was significantly greater during the inconsistent condition compared to the consistent condition. No significant interaction was observed between the group and the condition.

The results from the Cz electrode in the 200–500 ms time range showed a significant main effect of group [*F* (1, 34) = 10.06, *p* = 0.0026, *η^2^p* = 0.23]. The oscillatory activity in the HPAL group was significantly higher than that in the LPAL group (Figure 3B). The main effect of condition and the interaction between group and condition were not statistically significant. At the Pz electrode, the analysis of theta oscillations indicated that, within the 0–1,000 ms time window, neither the main effects of group and condition nor their interaction were significant.

### 3.4 Alpha oscillations

In the analysis of the Fz electrode, the results indicated a significant effect of group within the time ranges of 0-200 ms [*F* (1, 34) = 10.14, *p* = 0.001, *η^2^p* = 0.23] and 200-500 ms [*F* (1, 34) = 8.05, *p* = 0.0059, *η^2^p* = 0.19]. As shown in Figure 3A, the LPAL group demonstrated a greater desynchronization of alpha oscillations compared to the other group. However, the main effect of condition was found to be insignificant within the 0–1,000 ms time window, and no significant interaction between group and condition was observed.

Regarding the Cz electrode, a significant main effect of group on alpha oscillations was detected in the 200-500 ms time window [*F* (1, 34) = 4.59, *p* = 0.0313, *η^2^p* = 0.19]. The oscillatory activity in the HPAL group was significantly higher than that in the LPAL group. Again, in the 0–1,000 ms time window, neither the main effect of condition nor the interaction between group and condition reached significance. At the Pz electrode, a significant main effect of group on alpha oscillations was observed in the 200-500 ms time window [*F* (1,34) = 5.91, *p* = 0.0146, *η^2^p* = 0.14]. The alpha frequency band oscillatory activity in the LPAL group was significantly lower than that in the HPAL group, as illustrated in Figure 3C. Additionally, there was no significant main effect of condition nor any significant interaction between group and condition within the 0–1,000 ms time window.

### 3.5 Correlation between physical activity and the amplitudes of P300 and oscillations

Spearman correlation was utilized to assess the relationship between physical activity levels and the amplitude of P300. At the Fz electrode, a significant positive correlation was found between PA levels and the amplitude of P300 [*r* (36) = 0.35, *p* = 0.003]. Similarly, at the Cz electrode, the analysis revealed a significant correlation between the amplitude of P300 and PA levels [*r* (36) = 0.33, *p* = 0.004]. These findings suggest that higher PA levels among participants are associated with increased amplitudes of P300.

We also examined the correlations between PA levels and theta oscillations. At the Fz electrode, a significant correlation was observed between PA and theta oscillations in the 0–200 ms [*r* (36) = 0.31, *p* = 0.008] and 200–500 ms time windows [*r* (36) = 0.42, *p* < 0.001]. This indicates that individuals with higher PA levels tend to exhibit stronger theta oscillations. At the Cz electrode, the Spearman correlation analysis also indicated significant associations between PA and theta oscillations in the 200–500 ms time window [*r* (36) = 0.25, *p* = 0.035].

Moreover, at the Fz electrode, significant associations were found between PA levels and alpha oscillations in the 0–200 ms [*r* (36) = 0.41, *p* < 0.001] and 200–500 ms time windows [*r* (36) = 0.41, *p* < 0.001]. This suggests that participants with higher PA levels demonstrated stronger alpha oscillations during these periods. Additionally, significant correlations were found for alpha oscillations in the 200–500 ms time window at the Cz electrode [*r* (36) = 0.37, *p* = 0.001], indicating that greater PA levels are associated with enhanced oscillatory responses. Finally, at the Pz electrode, significant correlations were observed in alpha oscillations during the 200–500 ms time window [*r* (36) = 0.27, *p* = 0.024].

## 4 Discussion

This study found that the condition significantly impacted both RT and ACC during the task performance. Specifically, participants had notably shorter reaction times in the consistent condition compared to the inconsistent condition. Additionally, accuracy rates were significantly higher in the consistent condition than in the inconsistent one. These findings are consistent with previous research, which indicates that when the task was more complicated, it required more cognitive resources (Mehren et al., 2019). The results interestingly showed no differences between the groups with high and low levels of physical activity. This finding aligns with the outcomes of several studies (Goenarjo et al., 2020; van der Sluys et al., 2022). One possible explanation for this observation is that young individuals generally demonstrate stronger executive control abilities (Friedman et al., 2009). It may be the case that unless the cognitive demands of the experimental task are sufficiently high, the benefits of daily physical activity on inhibitory control are not apparent at the behavioral level (Chang et al., 2015; Kamijo et al., 2007). The flanker task employed in this study has relatively low cognitive requirements, which could account for the lack of significant differences between the two groups.

Although no significant behavioral differences were observed between the two groups with varying levels of physical activity, we identified notable differences in brain activity, which may indicate some underlying cognitive processes. Numerous studies have demonstrated that changes in neural activity may occur before or independently of changes in behavioral performance (Neubauer and Fink, 2009). For instance, individuals with high cognitive abilities often exhibit lower levels of cortical activation or greater neural efficiency when performing the same behavioral tasks. The enhanced theta and alpha power and the increased amplitude in P300 may indicate an optimized reorganization of specific neural networks. However, the current behavioral task may not be sensitive enough to detect such optimization. Similar phenomena are observed in skill learning studies, where changes in EEG theta band power can predict future improvements in behavioral performance, even though there are no noticeable differences in early behavioral indicators (Bönstrup et al., 2019).

The results revealed a significant group effect on the average P300 amplitude at the relevant electrodes. P300 is a well-studied, stimulus-locked component whose amplitude reflects the amount of attentional resources allocated to a specific task. In the current study, during the flanker task, the amplitude of P300 was significantly higher in the HPAL group compared to the LPAL group. Furthermore, the PA level of the participants was significantly correlated with the amplitude of P300. These findings support previous research (Aly and Kojima, 2020a,b; Lennox et al., 2019), suggesting that regular physical activity may enhance an individual’s ability to effectively allocate cognitive resources, enabling them to mobilize more cognitive power while performing tasks. Previous studies have shown that P300 is related to attention and has been used as an electrophysiological indicator of executive function (Hillman et al., 2004; Scharinger et al., 2017). Some studies indicate that when performing motor tasks, the group with higher levels of exercise exhibits a larger P300 amplitude compared to the group with lower levels of exercise (Li et al., 2024; Olson et al., 2023; Smith et al., 2013). This suggests that exercise can enhance cognitive function by increasing the allocation of attentional resources. Pontifex and Hillman observed similar results, noting an increase in P3 amplitude during movement, which they interpreted as a relative inefficiency of neural electrical resources during physical activity (Pontifex and Hillman, 2007).

The N200 is a specific brain component linked to inhibitory control processing. Numerous studies have indicated that this N200 component primarily reflects the processes and intensity involved in conflict monitoring and inhibition (Enriquez-Geppert et al., 2010; Johnstone and Galletta, 2013). However, some previous research has questioned the validity of the flanker N200 as a reliable measure of response conflict (Tillman and Wiens, 2011). In a study that using a large-sample ERP study employing a basic flanker task, they did not find evidence for the conflict N2 component (Kałamała et al., 2018). In the current study, no significant changes in the amplitude of N200 were observed between the conditions and the groups, which is in line with the previous research (Hsieh et al., 2018; Kałamała et al., 2018).

According to the time-frequency analysis, the results indicated a significant main effect of the group on theta oscillation activity. Specifically, the theta oscillation activity in the HPAL group was significantly higher than that in the LPAL group. This finding aligns with previous research showing that theta oscillation activity is greater in exercise groups compared to control groups (Shukla et al., 2020). One explanation for this phenomenon is that physical activity enhances the overall activation of the nervous system, influencing neural oscillations across the brain (Ciria et al., 2018). Another perspective suggests that theta oscillation activity reflects an individual’s allocation of attention and engagement in their cognitive environment, implying that increased physical activity can improve attention allocation capabilities (Griggs et al., 2023; Pastötter et al., 2013). In the current study, we found that theta oscillation activity was significantly higher in the inconsistent condition compared to the consistent condition. Previous research has shown that theta power amplitude is sensitive to the activation of executive control in interference situations. The increased theta power observed in the inconsistent condition may be associated with a greater recruitment of cognitive control to manage conflicts during information processing. This could reflect a greater investment of neural resources to suppress interference (Cavanagh and Frank, 2014).

Significant differences in alpha oscillations were observed among participants based on their levels of physical activity. Individuals with low physical activity levels showed a notable desynchronization of alpha oscillations compared to those with high physical activity levels. Furthermore, we found a significant positive correlation between physical activity levels and alpha oscillations. These findings align with previous studies. One review indicates that increased alpha-band activity is frequently linked to exercise, as shown in various studies (Hosang et al., 2022). Additionally, alpha waves were found to play a mediating role in the relationship between the duration of physical activity and the executive function of children (Arabi et al., 2023). It is suggested that higher alpha amplitude indicates functional inhibition in the cerebral cortex (Klimesch et al., 2007) and is closely related to attention allocation (Jia et al., 2019). One possible explanation for the observed differences is that participants in the high-activity group possess better attentional control, enabling them to focus more effectively on target information. In contrast, insufficient alpha oscillation activity may be linked to cognitive deficits or a lack of inhibitory control (Knyazev, 2007), which could indicate that the low-activity group may struggle with inhibitory control. Overall, our findings suggest that increased alpha activity may be associated with enhanced inhibitory control in individuals with higher levels of physical activity.

## 5 Conclusion

This study utilized EEG technology and the Flanker paradigm to investigate how daily physical activity levels affect inhibitory function, examining aspects such as behavior, ERP components, and neural oscillations. The results indicated that engaging in daily physical activity can enhance inhibitory function. Specifically, individuals with high physical activity levels demonstrated stronger inhibitory function compared to those with low physical activity levels. This improvement was primarily reflected in an increased amplitude of P300, as well as heightened theta and alpha oscillation activity. These findings suggest that daily physical activity can improve an individual’s capacity to allocate attention resources, allowing them to mobilize more cognitive resources when performing tasks that require inhibition.

## Data Availability

The raw data supporting the conclusions of this article will be made available by the authors, without undue reservation.
